# Biosynthesis of the Tricyclic Aromatic Type II Polyketide Rishirilide: New Potential Third Ring Oxygenation after Three Cyclization Steps

**DOI:** 10.1007/s12033-021-00314-x

**Published:** 2021-03-24

**Authors:** Ahmad Alali, Lin Zhang, Jianyu Li, Chijian Zuo, Dimah Wassouf, Xiaohui Yan, Philipp Schwarzer, Stefan Günther, Oliver Einsle, Andreas Bechthold

**Affiliations:** 1 Institute of Pharmaceutical Biology and Biotechnology, Albert-Ludwigs-Universität, Stefan-Meier-Straße 19, 79104 Freiburg, Germany; 2 Institute of Biochemistry, Albert-Ludwigs-Universität, Albertstr 21, 79104 Freiburg, Germany; 3 Institute of Pharmaceutical Bioinformatics, Albert-Ludwigs-Universität, Hermann-Herder-Str 9, 79104 Freiburg, Germany

**Keywords:** Rishirilide, Cyclization, Oxygenation, Epoxidation, CRISPR-Cas9

## Abstract

Rishirilides are a group of PKS II secondary metabolites produced by *Streptomyces bottropensis* Gö C4/4. Biosynthetic studies in the past have elucidated early and late steps of rishirilide biosynthesis. This work is aiming to solve the remaining steps in the rishirilide biosynthesis. Inactivation of the cyclase gene *rslC3* in *Streptomyces bottropensis* resulted in an interruption of rishirilide production. Instead, accumulation of the tricyclic aromatic galvaquinones was observed. Similar results were observed after deletion of *rslO4*. Closer inspection into RslO4 crystal structure in addition to site-directed mutagenesis and molecular dynamic simulations revealed that RslO4 might be responsible for quinone formation on the third ring. The RslO1 three-dimensional structure shows a high similarity to FMN-dependent luciferase-like monooxygenases such as the epoxy-forming MsnO8 which acts with the flavin reductase MsnO3 in mensacarcin biosynthesis in the same strain. The high sequence similarity between RslO2 and MsnO3 suggests that RslO2 provides RslO1 with reduced FMN to form an epoxide that serves as substrate for RslO5.

**Supplementary Information:**

The online version contains supplementary material available at 10.1007/s12033-021-00314-x.

## Introduction

Rishirilide is a PKS-II secondary metabolite isolated from two soil inhabitant Streptomyces species [*S. rishiriensis* OFR-1056 and *S. bottropensis* Gö C4/4 (*S. bottropensis*)] and one deep-sea species (*S. olivaceus* SCSIO T05) [1, 2]. This compound has been shown to have moderate biological activities including the inhibition of α2-macroglobulin, glutathione S-transferase, and asparagine-tRNA synthase, whose roles in cancer and thrombosis have been sufficiently characterized over the past years [3].

Structurally, rishirilide is a tricyclic partially aromatic polyketide with an unusual isopentyl sidechain and a carbonyl group on the third ring. This makes it an intriguing target of the biosynthetic pathway studies. Schwarzer et al. identified the origins of the tricyclic backbone and some other substitutions in rishirilide B. The analysis of labelled compound using Nuclear Magnetic Resonance (NMR) spectroscopic revealed that the origin of isopentyl sidechain is isobutyryl-CoA, which is derived from valine. The tricyclic skeleton is synthesized from nine acetate units, and the methyl group is derived from a decarboxylation of one acetate unit [4].

In the course of our rishirilide biosynthetic pathway studies, a remarkable amount of information on early and tailoring steps has been recently elucidated and published. The polyketide synthase RslK4 and the putative 3-oxoacyl-ACP reductase RslO3 are involved in the generation of the unusual starter unit, and the early acting ketoreductase RslO10 is involved in first ring cyclization/aromatization [5].

Gene inactivation studies and biological assays yielded a detailed insight into the rearrangement of the oxidative carbon backbone in rishirilide biosynthesis. The flavin-dependent RslO5 is responsible for epoxide-opening in its anthraquinonic substrate. Subsequently, flavin monooxygenase RslO9 mediates the lactone-forming Baeyer–Villiger oxidation, and the resulted rearranged intermediate is converted to rishirilide A via the ketoreductase RslO8. Rishirilide A is then converted to rishirilide B via RslO9 and water release [6].

*S. albus* containing a cosmid with genes of the rishirilide biosynthetic gene cluster (cos4) was producing rishirilide B as major compound. *S. albus* containing cos4 with a deletion in *rslO4* was producing galvaquinone A and B. And *S. albus* containing cos4 with a deletion in *rslO1* was producing galvaquinone A as major compound. We have proposed that galvaquinone A and B are shunt products resulting from unstable intermediates [5].

In this study the involvement of one cyclase and two oxygenase in rishirilide biosynthesis was investigated. For this purpose, excision of *rslC3* gene using the markerless time-saving CRISPR-Cas9 system, inactivation of *rslO4* gene using Redirect® technology, and crystallization of RslO4 and RslO1 proteins were carried out. Molecular dynamics simulations and site-directed mutagenesis were used to predict the roles of RslO4 and RslO1 in quinone formation and epoxidation on the third ring, respectively.

## Materials and Methods

### Bacterial Strains

*S. bottropensis* and *Streptomyces albus* J1074, *E*. *coli* DH5α for plasmid propagation, and *E*. *coli* ET12567/pUZ8002 for intergeneric conjugation were used. Long-term storage of *Streptomyces* and *E. coli* cells was done in glycerol 15% *(V/V)* at – 20 °C and − 80 °C, respectively.

### Bacterial Cultivation and Expression Media

TSB medium (Caso Boullion 30 g/l) for *Streptomyces* cultivation. LB-medium (Luria Bertani 20 g/l) and LB-agar medium (LB medium with agar–agar 21 g/l) for *E*. *coli* strains cultivation. SG^+^ medium (glucose 20 g/l, soytone 10 g/l, CaCO_3_ 2 g/l, CoCl_2_ 0.1% m/V, L-valin 2.34 g/l) for secondary metabolites production in *S. albus* strains. YmPG medium (yeast extract 4 g/l, pepton from casein 1 g/l, glucose 10 g/l, malt extract 10 g/l, MgCl_2_.6H_2_O 2 g/l, L-valin 5% 40 ml/l) for secondary metabolites production in *S. bottropensis* strains. MS-agar medium (mannitol 20 g/l, soya flour 20 g/l, agar–agar 21 g/l, MgCl_2_ 10 mM) for intergeneric conjugation. 2 × YT medium (tryptone 16 g/l, NaCl 5 g/l, yeast extract 10 g/l) for heat shock of *S. bottropensis* spores preparing for intergeneric conjugation. All media were freshly prepared, initial pH values were adjusted to 7.2 and directly autoclaved (Tuttnauer Systec 5075 ELV, Germany).

### CRISPR-Cas9 System for Gene Deletion of *rslC3* in *S. bottropensis*

The system used in this study has been developed and optimized for actinomycetes in The Novo Nordisk Foundation Center for Biosustainability [7]. Two cloning procedures were required to prepare each construct: cloning of single-guide RNA (sgRNA) scaffold specific for the target gene between NcoI and SnaBI restriction sites and cloning of Homologous-Directed Repair Template (HDR Template) into StuI restriction site. sgRNA scaffold was designed by fusing CRISPR RNA (crRNA, 20 nt specific to a unique sequence in the target gene) with the associated sgRNA core sequence (trans-activating CRISPR RNA, tracr RNA). CRISPOR tool offered online from Zhang Lab was used for detecting and evaluating the 20 nt crRNA guide sequences ending with—NGG as Protospacer Adjacent Motifs (PAM) for targeting *rslC3*. In order to prepare the HDR-Template, 800 bps upstream—and 800 bps downstream the target gene was PCR-amplified with 40 complementary base pairs from the sides to be connected, then the two PCR products were joined by overlapping PCR technique using only the two primers annealed from the other sides (Table S1).

To transform the constructs into *S. bottropensis*, *E*. *coli* ET12567/pUZ8002 was grown to an OD_600_ = 0.4–0.6 in the presence of apramycin (50 µg/mL) and kanamycin (50 µg/mL). After centrifugation cells were washed and mixed with 50 °C heat-shocked spores on MS-agar plates supported with MgCl_2_ (10 mM) at 28 °C for 16–20 h. After that, the plates were overlaid with apramycin (50 µg/mL) and fosfomycin (200 µg/mL) and incubated again at 28 °C until the exconjugants appeared. During replica-plating of the exconjugants, endonuclease activity of Cas9 was induced by thiostrepton (1 µg/mL). Finally, deletion of the target gene in the genomic DNA of some exconjugants was verified by PCR and sequencing.

### Complementation of *ΔrslC3* Mutant

After deletion of *rslC3* gene in *S. bottropensis*, the temperature sensitive plasmid was eliminated by growing at 37 °C in liquid TSB media till the cells lost the resistance to apramycin. Following this, *S. bottropensis—∆rslC3* spores were transformed with the integrative expression vector pSET152 harboring *rslC3* gene (cloned between XbaI and EcoRV restriction sites) by intergeneric conjugation taking into consideration that pSET152 vector carries apramycin resistance marker (Table S1).

### Redirect® Technology for *rslO4* Gene Editing in *S. bottropensis*

The protocol used for this study has been developed by Gust B, Kieser T, and Chater KF [8]. The procedure was adapted to this work with some modifications. For this purpose, Cosmid 4 prepared in our laboratory by Xiaohui Yan from pOJ436 backbone with the whole gene cluster of rishirilide (Rsl gene cluster) was used after replacing the integrase gene with ampicillin^R^ marker by Olga Tsypik and replacing apramycin^R^ marker with spectinomycin^R^ marker in this work. The gene *rslO4* was replaced by apramycin cassette using homologous recombination in an *E*. *coli* strain containing pBADαβγ plasmid in the presence of L-arabinose (0.01 M). pLEERE and pCDF Deut-1 vectors were used as PCR templates for the amplification of apramycin^R^ and spectinomycin^R^ cassettes, respectively. Forward and reverse primers were designed as mentioned in the protocol from Gust and his colleagues with 60 nt (40 nt complementary to the flanking regions of the target gene and 20 nt corresponding to the cassette sequence). PCR amplification method mentioned in the protocol was followed as well. After replacing *rslO4* gene with apramycin^R^ cassette, the cosmid was transformed into *S. bottropensis* by intergeneric conjugation using *E*. *coli* ET12567/pUZ8002 strain. The overnight growing *E*. *coli* cells containing the prepared non-integrative *rslO4*-mutant Cos4 in the presence of apramycin (50 µg/mL), spectinomycin (100 µg/mL), and kanamycin (50 µg/mL) were mixed with the mycelia on MS-agar supported with MgCl_2_ (10 mM) at 28 °C for 8 h. Then, the plates were overlaid with apramycin (50 µg/mL) and fosfomycin (200 µg/mL) but not with spectinomycin because the cosmid was suicidal after undergoing double cross-over with *S. bottropensis* genomic DNA. Replacement of *rslO4* in the genomic DNA of *S. bottropensis* with apramycin cassette was verified by PCR depending on the difference in length between *rslO4* (300 bps) and apramycin cassette (1045 bps) (Table S1).

### Induction of Secondary Metabolites Production in *S. bottropensis*∆*rslO4* by Positive Regulators

We recently described that *S. albus* J1074 containing cos4 and a plasmid containing all three regulatory genes *rslR1*, *rslR2* and *rslR3* produced more rishirilide B than *S. albus* J1074 containing only cos4 [5]. In an attempt to find the natural substrate of RslO4 enzyme, the Δ*rslO4* mutant of *S. bottropensis* was transformed with the replicative vector pUWL-H harboring the three regulatory genes *rslR1*, *rslR2* and *rslR3.* Intergeneric conjugation and overlay with apramycin (50 µg/mL), hygromycin B (50 µg/mL), and fosfomycin (200 µg/mL) were carried out.

### Expression and Extraction of Secondary Metabolites of Streptomyces

YmPG and SG^+^ media were used in this study for the fermentation and secondary metabolite production in *S. bottropensis* and *S. albus* strains, respectively. Prior to fermentation, precultures of the strains were prepared in TSB medium in the presence of appropriate antibiotics at 28 °C/180 rpm in the shaking incubator (Infors HT, Switzerland) for 2–3 days till the formation of well-growing cultures. The fermentation medium was inoculated with 1% of the preculture and then incubated at 28 °C/180 rpm for 5 days. Extraction of the secondary metabolites was carried out by ethyl acetate at pH 4, subsequently ethyl acetate was evaporated, and the crude extracts were solved in methanol (100%) and analyzed by HPLC/ESI–MS (Table S2).

### Overexpression and Purification of RslO1 and RslO4 Proteins

Each of *rslO4* and *rslO1* genes were amplified using cosmid 4 as a template. The amplified PCR products were cloned into the expression vector pET21a(+) (Novagen, Merck, Darmstadt, Germany) between NdeI and XhoI restriction sites to produce C-terminal His-tag recombinant proteins (Table S1). The resulted constructs were verified by PCR and DNA sequencing. Expression was performed in *E. coli* BL21 (DE3) Star cells (Invitrogen, Thermo Fisher, Waltham, USA). The precultures of *E. coli* BL21 (DE3) cells containing the constructs were prepared in LB-media at 37 °C/180 rpm in the presence of 100 µg/ml ampicillin overnight. Thereafter, the growing cells were inoculated in 3 L LB-media for each protein and incubated at the same conditions. By reaching an OD_600_ of 0.6, the overexpression was induced by addition of isopropyl β-D-1-thiogalactopyranoside (IPTG, Carl Roth, Karlsruhe, Germany) in a concentration of 0.1 mM and the media were incubated again at 20 °C/180 rpm for 12 h. Cells were harvested 12 h after induction by Centrifugation at 5000 × *g* for 10 min (Rotina 380P, Hettich, Germany). The cell pellet was re-suspended in four pellet volumes of lysis buffer [Tris–HCl (pH 8.0, 50 mm), NaCl (300 mm)]. The cells were lysed using French Pressure cell (40 K) at a pressure of 800 to 1000 psi. The suspension was mixed with 10 μL of DNase and incubated for 15 min on ice. Thereafter, the protein containing soluble fraction was separated by centrifugation at 10,000 × *g*, 4 °C, 1 h (Avanti J-6000 Rotor JA-10, Bechmann Coulther, Germany). This fraction was further purified by IMAC Ni–NTA affinity chromatography as follows: The protein sample was loaded on a 5 mL HisTrap™ FF column (GE Healthcare GmbH, Solingen, Germany), which was previously equilibrated with lysis buffer, using a flow rate of 0.5 ml/min. The column was then stepwise washed with 5–15% elution buffer [Tris–HCl (pH 8.0, 50 mm), NaCl (300 mm), imidazole (500 mm)] at a flow rate of 2 ml/min. The desired protein was eluted with 50% elution buffer, analyzed by SDS-PAGE and its stability was observed. Fractions containing the desired protein were pooled and concentrated to a final volume of 1 mL by centrifugation in a Vivaspin concentrator (Sartorius, Göttingen, Germany). Finally, the protein was purified with a size exclusion HiLoad® 16/60 Superdex® 200 column (GE Healthcare, Solingen, Germany), pre-equilibrated with buffer [Tris–HCl (pH 7.5, 20 mm), NaCl (150 mm)].

### Crystallization and Structure Determination of RslO1

After size exclusion chromatography (Superdex S200 16/60) in buffer (20 mM Tris pH 8.0, 150 mM NaCl), RslO1 protein was concentrated to 24 mg/ml before setting up initial screens using sitting drop vapor-diffusion method at 20 °C. A drop of 0.6 µl in total with different protein to reservoir ratios (25%, 50%) was set and equilibrated again reservoir solutions (50 µl), using an automatic crystallization drop-set system (Oryx Nano, Douglas Instrument). Large crystal clusters were grown for four weeks in 0.2 M calcium chloride, 0.1 M HEPES pH 7.0 and 33% polyethylene glycol conditions. Then these crystals were crashed for seeding experiment by using Oryx Nano. Then nice single crystals were grown in conditions with 0.1 M HEPES pH 7.5 and 36% polyethylene glycol 600 after one week and kept growing for 4 weeks before harvesting. Crystals were mounted on cryo-loops and flash frozen in liquid nitrogen prior to data collection. Datasets to 1.6 Å were collected at beamline X06SA (PXI) at Swiss Light Source (Villigen, Switzerland) with the PILATUS 6 M detector. The data were processed using iMosflm [9] and XDS package [10], the crystal was assigned to the space group P2_1_2_1_2_1_ with unit cell dimensions *a* = 50.89, *b* = 81.77 Å and *C* = 160.61 Å.

RslO1 monooxygenase crystal structure was determined by automated molecular replacement in MrBUMP [11] using monomer of a monooxygenase from *Agrobacterium tumefaciens* (PDB code: 2I7G, amino acid sequence identity of 24%) as search model. Automated model building was carried out with Buccaneer [12], and manual model building was performed in COOT [13]. Refinement of the initial electron density was carried out using cycles of the program phenix.refine [14]. The final structure was refined to *R*_cryst_ = 18% and *R*_free_ = 20% at resolution of 1.60 Å. The quality of the structure was validated by MOLPROBITY [15]. (Table S3).

### Crystallization and Structure Determination of RslO4

After size exclusion chromatography (HiLoad Superdex S75 26/60, Merck, Darmstadt, Germany) in buffer solution (Tris/HCl (pH 8.0) 20 mm, NaCl 150 mm), the protein was diluted to 20 mg/ml. Initial screens were set up using the sitting drop vapor-diffusion method at 20 °C. A drop of 0.6 µL total volume with different protein to reservoir ratios (1:3, 1:1, 3:1) was set with an automated crystallization drop-setter (Oryx Nano, Douglas Instrument, Berkshire, UK) and equilibrated against different reservoir solutions (50 µL). Large single crystals appeared within two days with a reservoir condition containing 6% ethanol, 0.1 m ammonium acetate (pH 5.5) and 1.5 m phosphate (pH 7.0). Crystals were mounted in nylon loops and flash-frozen in liquid nitrogen prior to data collection. A dataset to 1.8 Å resolution was collected at beamline X06DA of the Swiss Light Source (Villigen, Switzerland) with a PILATUS 2 M detector. The data were processed with autoPROC, and the crystal belonged to space group *P*3_2_21, with unit cell dimensions of *a* = *b* = 83.7 Å and *c* = 82.3 Å [16]. The asymmetric unit contained two monomers, at a solvent content of 47.43%. The crystal structure of RslO4 was determined by molecular replacement in MOLREP using the monomer of SnoaB (PDB: 3KNG, amino acid sequence identity of 41%) as search model [17]. Refinement of the initial electron density was carried out by iteratively using phenix.refine, followed by model building in COOT [13, 14]. The final structure was refined to *R*_cryst_ = 0.18 and *R*_free_ = 0.20 at a resolution of 1.8 Å. The geometry of the structure was validated by MOLPROBITY [15].

### In Vivo Comparison of RslO4 and SnoaB Activities

To compare between RslO4 and SnoaB enzymatic activity in the oxygenation of RslO4-substrate and reproducing rishirilide B, each of encoding genes was cloned with replicative expression vector pUWL-H between ClaI and SpeI restriction sites and transferred into *S. albus* × Cos4Δ*rslO4* growing mycelia by intergeneric conjugation on MS-agar supported with MgCl_2_ (10 mM) using *E*. *coli* ET12567/pUZ8002 cells. After 8 h incubation at 28 °C, the plates were overlaid with apramycin (50 µg/mL), hygromycin B (50 µg/mL), and fosfomycin (200 µg/mL) and incubated again at 28 °C till the exconjugants appeared. The secondary metabolites were produced in SG + media at 28 °C/180 rpm for 5 days, then the crude extracts were extracted by ethyl acetate at pH 4 and analyzed by HPLC/ESI–MS (table S2).

### Side-Directed Mutagenesis (SDM) Inside the Binding Cavity of RslO4 Protein

The whole pUWL-H-*rslO4* plasmid was PCR-amplified using back-to-back 5´-phosphorylated primers. At 5′-end, forward primers were designed with alanine codon for the substitution mutation of asparagine 7, asparagine 52 or methionine 29, and with phenylalanine codon for the substitution mutation of tryptophan 56 or tyrosine 22 (Table S1). In order to investigate the role of each amino acid in the binding to RslO4 substrate, the PCR products were self-ligated and transferred separately into *S. albus* × Cos4Δ*rslO4* strain using the protocol of intergeneric conjugation on MS-agar supported with MgCl_2_ (10 mM) using *E*. *coli* ET12567/pUZ8002 cells. After 8 h incubation at 28 °C, the plates were overlaid with apramycin (50 µg/mL), hygromycin B (50 µg/mL) and fosfomycin (200 µg/mL) and incubated again at 28 °C till the exconjugants appeared. The secondary metabolites were produced in SG + media at 28 °C/180 rpm for 5 days, then the crude extracts were extracted by ethyl acetate at pH 4 and analyzed by HPLC/ESI–MS (Table S2). The residual activity of RslO4 was measured depending on calculating the relative AUC of reproduced rishirilide B in the crude extracts of *S. albus* × Cos4Δ*rslO4* complemented with mutants RslO4 in Asn7, Asn52, Trp56, Tyr22 and Met29 comparing to relative AUC of reproduced rishirilide B in the crude extract of *S. albus* × Cos4Δ*rslO4* complemented with wild type RslO4.

### In Silico Prediction of RslO4-Ligands Interactions

Molecular modelling was performed using the Schrödinger Software Suite (Schrödinger Release 2019–2). The structure of RslO4 was prepared using the Protein Preparation Wizard. In this step, atom types and bond orders were assigned, missing atoms were added, water molecules beyond 5 angstroms from hetero groups were deleted, water orientations were sampled, tautomer/ionization states were assigned corresponding to pH 7, the hydrogen bonding network was optimized, followed by restrained minimization using the OPLS3 force field. In the next step, all putative substrates were docked into the active site of RslO4 using Glide XP with flexible ligand sampling. All default settings were used except intramolecular hydrogen bonds were rewarded and planarity of conjugated pi groups was enhanced.

The molecular dynamics simulations for selected models were performed using the GPU-accelerated Desmond program within the Schrödinger Software Suite (Schrödinger Release 2019–2). The SPC solvent model was used, and the force field was set to OPLS3e. The system energy was set to 1.2, and NPT was used as the ensemble class. All systems were relaxed before simulations. Simulations were set to run at 300.0 K and at 1.01325 bar. Simulation time for the putative substrate was set to 35 ns with 35 ps trajectory recording intervals, and simulation time for the putative intermediate was set to 70 ns with 70 ps trajectory recording intervals, resulting in an MD trajectory with 1000 frames per simulation.

## Results

### Sequence Analysis of RslC1, RslC2, and RslC3 Proteins

Sequence analysis using BLASTp was used to predict the functions of RslC1, RslC2, and RslC3 proteins. First, the putative first ring CYC/ARO of nogalomycin (SnoaE), rhodomycin (RdmK) and chartereusin (ChaF) were found to be homologs of RslC1 with 53%, 54% and 55% identity, respectively [18, 19]. Second, the homologs of RslC2 included ActIV (55% identity), AlnR (54%) and Ssfy2 (53%) that act probably as second ring CYC/ARO in the biosynthesis of actinorhodin, alnumycin and tetracycline SF2575, respectively [20–22]. Third, sequence alignments of RslC3 to the proposed fourth ring CYC/ARO in chromomycin (CmmX), oxytetracycline (OxyL) and nogalomycin (SnoO) showed 49%, 44% and 50% identity, respectively [23–25].

### Inactivation of *rslC3 and rslO4* in *S. bottropensis*

In order to show that *rslC3* is involved in rishirilide biosynthesis and in order to confirm the involvement of *rslO4* gene deletion experiments were carried out in *S. bottropensis*. Trace amounts of rishirilide were detected in the crude extract of *S. bottropensisΔrslC3*. In addition, the tricyclic aromatic galvaquinones (Fig. S1) could be detected in the *rslC3* mutant (galvaquinone 1 and 2). Complementation of the *rslC3* mutant led to an increase in rishirilide B production but formation of galvaquinones was also observed (Fig. S1), indicating that the biosynthetic machinery in the complemented mutant did not work as well as in the wt-strain. *S. bottropensis* with a deletion in *rslO4* also failed to produce rishirilides but accumulated galvaquinone 1 and 2 instead. In this mutant, galvaquinones production increased when a plasmid containing the positive regulatory genes *rslR1*, *rslR2* and *rslR3* were coexpressed (Fig. S2) resulting in a higher transcription of structural genes. The galvaquinones 1 and 2 were identical in their HPLC-retention times, UV- and Mass-spectra compared to galvaquinones A and B respectively. Structures of both compounds have been elucidated before as products of *S. albus* × Cos4Δ*rslO4* [5] (Supplementary Fig. S3). Based on UV- and MS spectra Galvaquinone 2 is identical to Galvaquinone B but its retention time in HPLC is different.

### Molecular Weight and Stability of RslO1 and RslO4

According to SDS-PAGE analysis, the molecular weights of C-terminal his-tagged proteins RslO1 and RslO4 were consistent with the predicted ones: 40.04 and 12.17 kDa, respectively. After 12 h on ice, no precipitation was observed, in addition to the stability by melting from freezing condition at − 80 °C to the room temperature.

### Crystal Structure of RslO4 (PDB ID:7BIO)

**Fig. 1:**
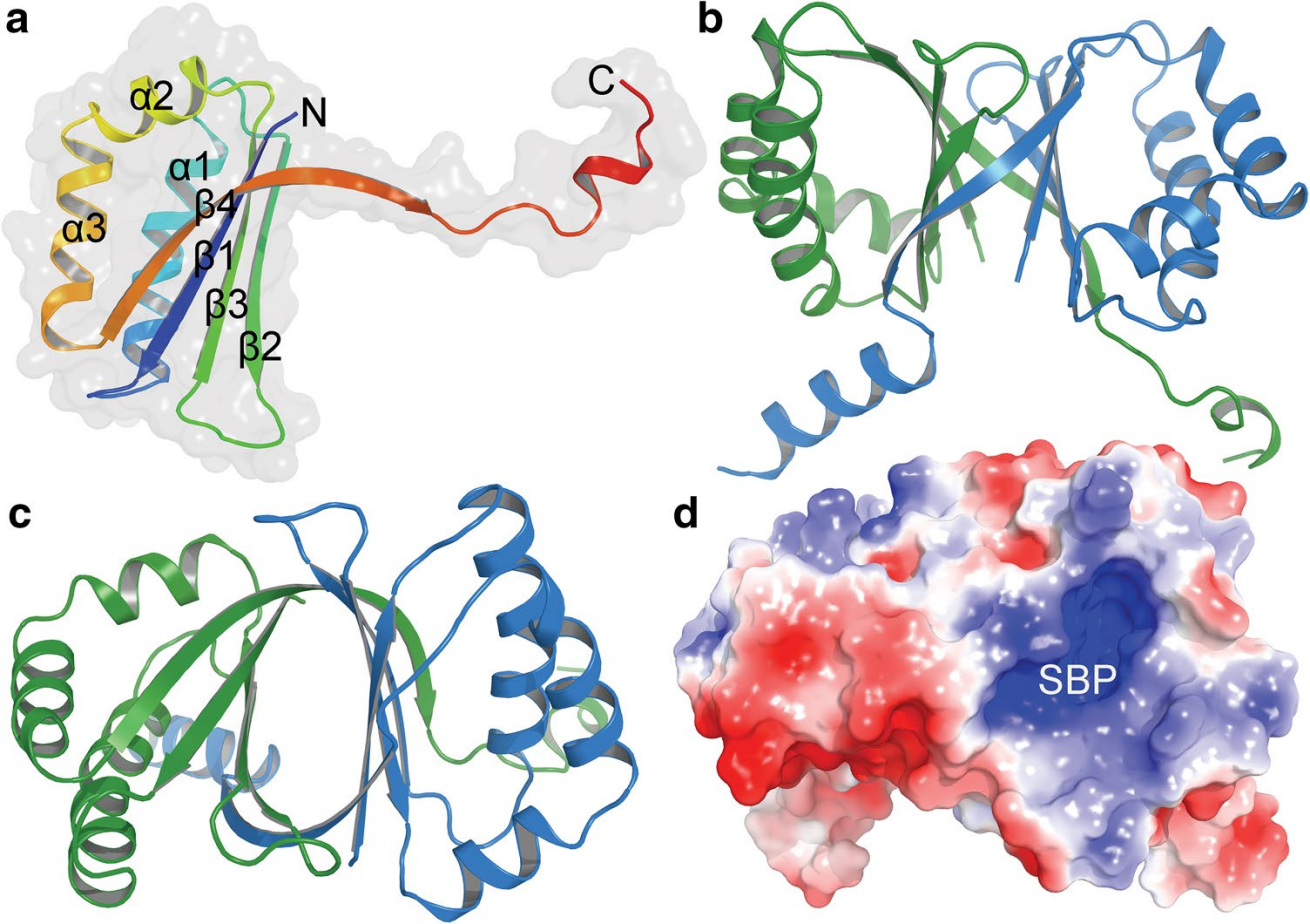
RslO4 Crystal structure: **a** Subunit A of RslO4 monooxygenase with the secondary structure elements labelled. The monomer contains three α helices (α1-α3) and four β strands (β1- β4) with a ferredoxin fold. The dimeric apo-form of RslO4 is shown from the side (**b**) and top view (**c**). The two subunits are coloured in green and blue, respectively. (**d**) Electrostatic potential surface shows a positively charged substrate binding pocket (SBP)

The monooxygenase RslO4 crystallizes as a homodimer and the overall structure of each subunit is made up of three α-helices (α1–α3) and four β-strands (β1–β4) with a ferredoxin-like fold (Fig. 1a). The additional α-helix in the C-terminus of each monomer is generated by the TEV protease cleavage site of the His-tag sequence, which is required for protein purification. The three α-helices are located on one side of the homodimer. Together with an additional β-strand of the other subunit, a five-stranded antiparallel β-sheet (Fig. 1b–d), which is stabilized by ten main-chain hydrogen bonds between β2 and β4 of two chains. This strand-swapping feature in dimer formation and stabilization can also be observed in homologous structures such as the monooxygenases ActVA-Orf6 from *Streptomyces coelicolor* and SnoaB from *Streptomyces nogalater*.

The interface between the two monomers is further stabilized by hydrophobic interactions provided by β-sheets and side chain hydrogen bonds contributed by Lys8, Ser43, Glu85 and His87. Data collection and refinement statistics are shown in table S3.

**Fig. 2:**
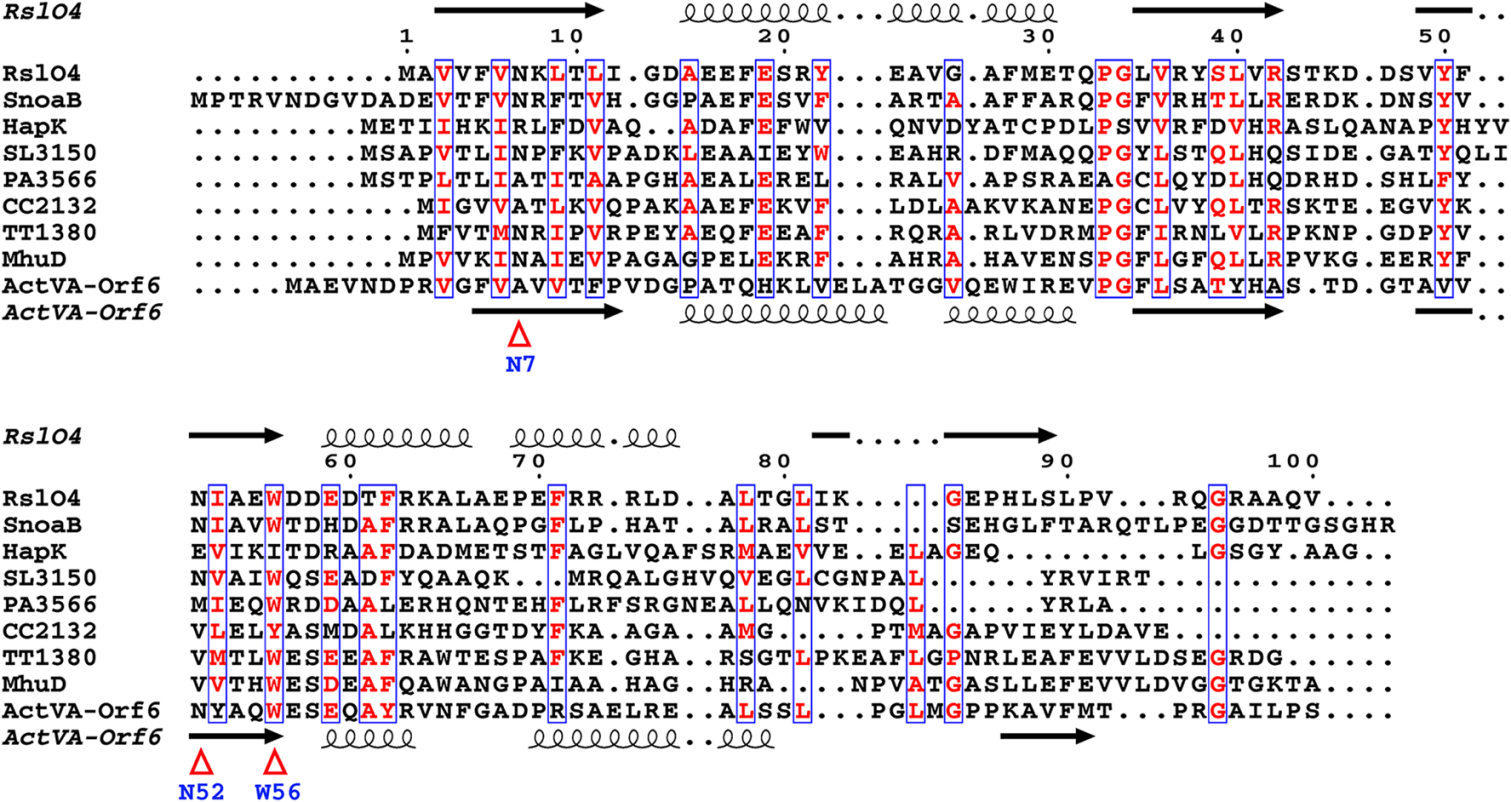
Sequence alignment of monooxygenases with the same fold like RslO4: The sequences have been selected based on identity from the output of SWISS-MODEL search, and the overall sequence identities between RslO4 and homologies are ranging from 16 to 41% [26]. SnoaB (UniProtKB AC: O54259) is a cofactor-independent monooxygenase from *S. nogalater* (PDB code: 3KNG) [27]. HapK (Q2S9J0) is a RedY-like protein from *Hahella chejuensis* KCTC 2396 (2JDJ) [28]. SL3150 (A3QHR8) is an antibiotic biosynthesis monooxygenase from *Shewanella loihica* (2RIL). PA3566 (Q9HY51) is an uncharacterized protein from *Pseudomonas aeruginosa* PAO1 (1X7V) [29]. CC2132(Q9A6G2) is an uncharacterized protein from *Caulobacter vibrioides* ATCC 19089 (3BM7). TT1380 (P83693) is from *Thermus thermophiles* HB8 (1IUJ) [30]. MhuD (P9WKH3) is a heme-degrading monooxygenase from *Mycobacterium tuberculosis* (3HX9) [31]. ActVA-Orf6 (Q53908) is a monooxygenase involved in the actinorhodin biosynthesis from *S. coelicolor* (1N5T) [32]. Sequence alignment was performed in ClustalW and the figure was prepared by ESPript 3.0 server [33, 34]

The closest structural homologue is SnoaB from *S. nogalater*, with a sequence identity of 41%. The second closest homologue, with an identity of 28% is SL3150, a monooxygenase from *Shewanella loihica* involved in antibiotic biosynthesis. All the homologues share a similar fold, even though the sequence identities for some of them are lower than 10%. The homologues are mostly uncharacterized and annotated as putative monooxygenases. Only a few have been biochemically investigated, including ActVA-Orf6 (of 16% identity), SnoaB and MhuD (of 22% identity). MhuD is a heme-degrading monooxygenase from *Mycobacterium tuberculosis*. It binds two heme groups, indicating the high flexibility of the substrate binding pocket. Sequence alignment of RslO4 together with the monooxygenases SnoaB and ActVA-Orf6, which both convert phenolic substrates to quinone products, showed that Asn52 and Trp56 are conserved in the negatively-charged active site (Fig. 2).

Closer inspection of the active site of RslO4 revealed high similarity between the positioning of Asn7, Asn52 and Trp56, which are represented by Asn18, Asn63 and Trp67 in SnoaB coordinating oxidized dithranol by hydrogen bonds. Comparing to ActVA-Orf6, positioning of Asn52 and Trp56, which are represented by Asn62 and Trp66 in ActVA-Orf6 coordinating oxidized acetyl dithranol by hydrogen bonds showed also high similarity. The hydrogen bond formed by Tyr51 in ActVA-Orf6 could be replaced by Tyr22 in RslO4.

**Fig. 3:**
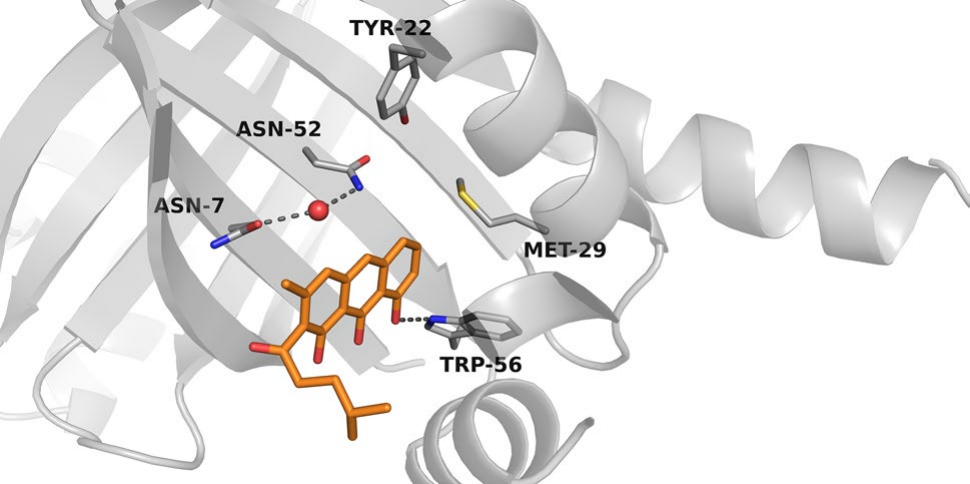
Predicted binding pose of the proposed tricyclic aromatic substrate inside RslO4 binding site

**Fig. 4:**
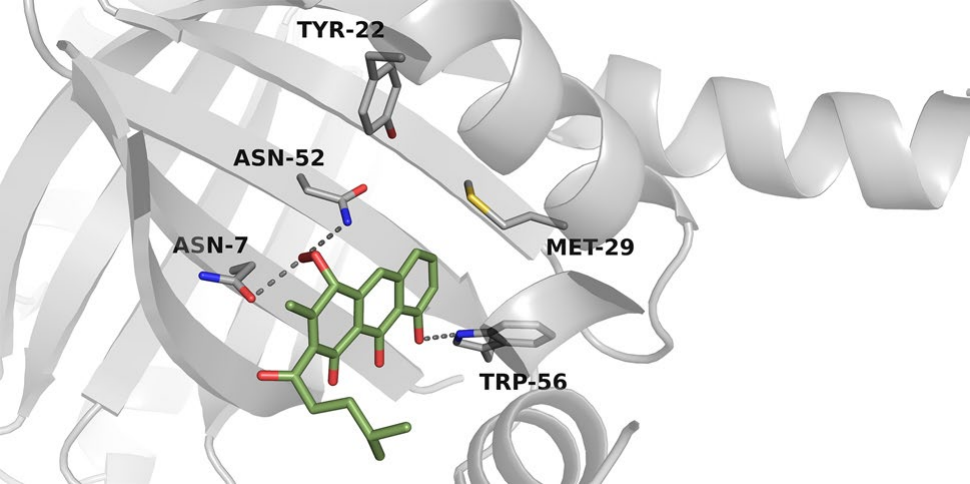
Predicted binding pose of the proposed peroxy intermediate inside RslO4 binding site

Observation from 35 ns molecular dynamic simulations suggested that the natural substrate of RslO4 is composed of tricyclic aromatic triol-anthracene structure with methyl group and methyl pentyl sidechain on the third aromatic ring. The Trp56 initiates a hydrogen bonding network to the three –OH groups (Fig. 3). On the hydrophobic side, Asn7-Asn52-Wat3 triad located close to the third aromatic ring. In 70 ns molecular dynamics simulations, this triad forms a hydrogen bond with the peroxide in the proposed intermediate of RslO4 (Fig. 4). It was also observed that Tyr22 contributes in the stabilization of peroxy intermediate inside the binding pocket.

### In Vivo Comparison of RslO4 and SnoaB Enzymatic Activities

In order to compare the monooxygenase activity of RslO4 with SnoaB, *S. albus* × Cos4∆*rslO4* was transformed with each of *rslO4* and *snoaB* genes cloned with replicative vector pUWL-H to test their enzymatic abilities in RslO4-substrate oxygenation and reproduce rishirilide B. Rishirilide B was successfully reproduced by complementation with *rslO4*. In contrast, no production of rishirilide B could be observed in *S. albus* × Cos4∆*snoaB.*

### Investigation on Oxygenation Mechanism of RslO4 by Site-Directed Mutagenesis

For deeper inspection of RslO4 catalytic machinery, substitution mutations in specific residues, which form the binding center, were created. Asparagine7, asparagine52 and methionine29 were replaced with alanine, while tryptophan56 and tyrosine22 were replaced with phenylalanine. Changes in *rslO4* gene sequence were performed in pUWL-H vector, thus it was possible to detect the incorporation of each residue in the enzymatic activity in rishirilide B biosynthesis in *S. albus* × Cos4∆*rslO4*. These results revealed that there is a clear trend of decreasing in rishirilide B production by the complementation of *S. albus* × Cos4∆*rslO4* with site-specific mutated *rslO4* genes comparing to the wild type. It was found that the substitution mutation of each of asparagine7 and asparagine52 reduced the production of rishirilide B by approx. 92%, whereas the replacement of tryptophan56 resulted in approx. 60% reduction. Creating substitution mutations of tyrosine22 and methionine29 showed the lowest influence on rishirilide B production (approx. 40% reduction in the activity) (Fig. S4).

### Crystal Structure of RslO1 (PDB ID: 7BIP)

**Fig. 5:**
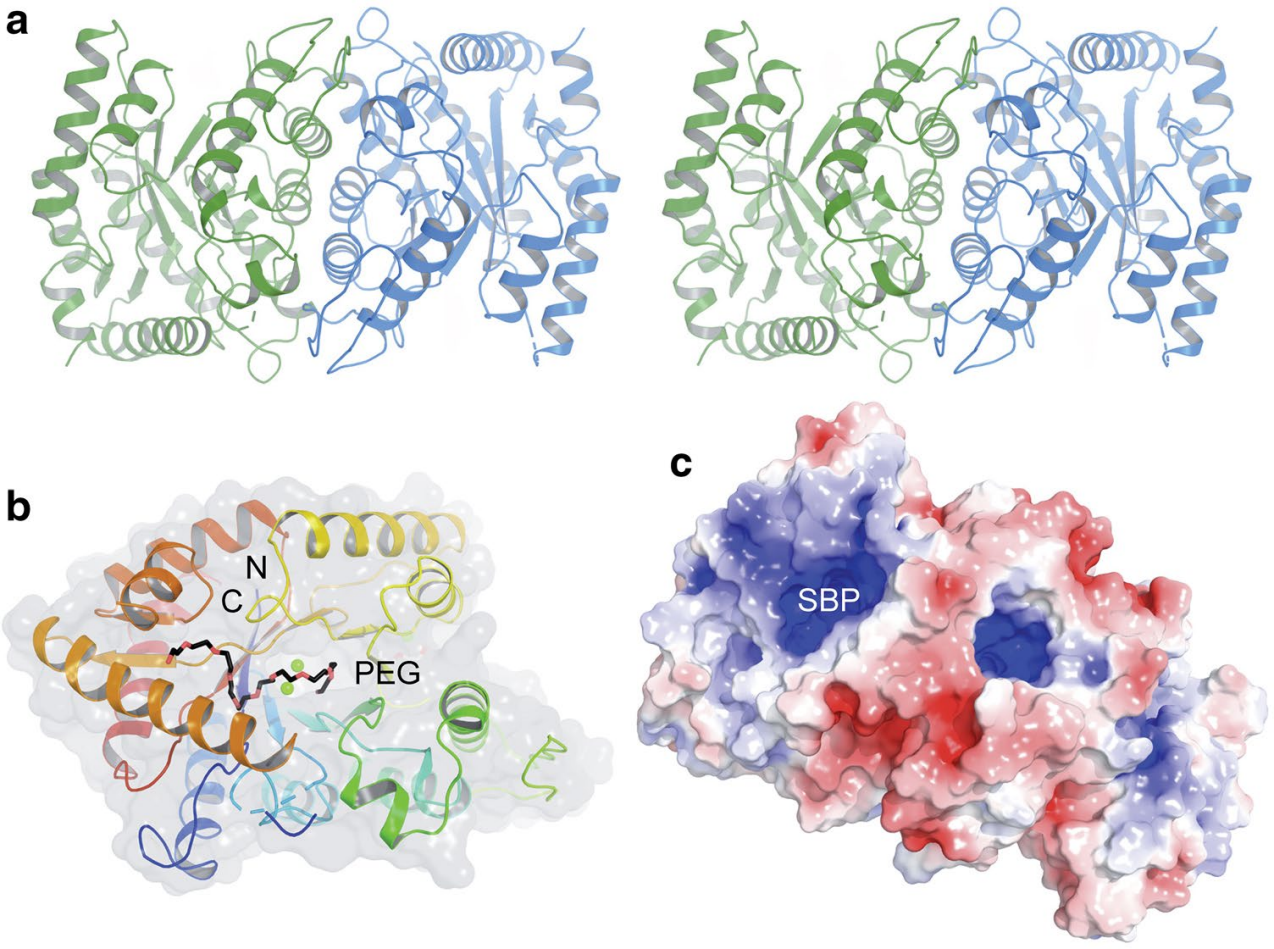
RslO1 crystal structure: **a** Stereo view of apo-form monooxygenase RslO1 structure. The two monomers form a tight homodimer. **b** In subunit A, the substrate binding site was occupied by a polyethylene glycol (PEG) molecule derived from the crystallization condition. **c** Electrostatic potential surface shows positively charged substrate binding pocket (SBP)

The monooxygenase RslO1 crystallizes as a homodimer and the overall structure of each subunit is made up of a well-conserved 8 β-strands TIM-barrel surrounded by 8 α-helixes. Each β-strand connects an α-helix through a short loop region. According to the structural homology study using Swiss Model Server, RslO1 was revealed to be FMN-dependent bacterial luciferase-like monooxygenase (Fig. 5). Data collection and refinement statistics are shown in table S3.

Structural homologs for RslO1 include MoxC (UnitprotKB AC: O34974) a putative monooxygenase from *B. subtilis* (PDB code: 1YW1); Lad A (A4IU28) an alkane monooxygenase from *G.thermodenitrificans* (3B9O) [35]; MeR (Q8TXY4) a 5,10-methylenetetrahydromethanopterin reductase from *M. kandleri* (1EZW) [36]; FgD1 (P9WNE1) a F420-dependent glucose-6-phosphate dehydrogenase from *M. tuberculosis* (3C8N) [37, 38]; AdF (O93734) a F420-dependent alcohol dehydrogenase from *M. thermophiles* (1RHC) [39]; SsuD (P80645) an alkanesulfonate monooxygenase from *E*. *coli* (1M41) [40]; LuxA (P07740) an alpha chain of an alkanal monooxygenase from *V.harveyi* (3FGC) [41]; and MsnO8 (W8QCH3), a luciferase-like monooxygenase from *S. bottropensis* (4US5) [42].

**Fig. 6:**
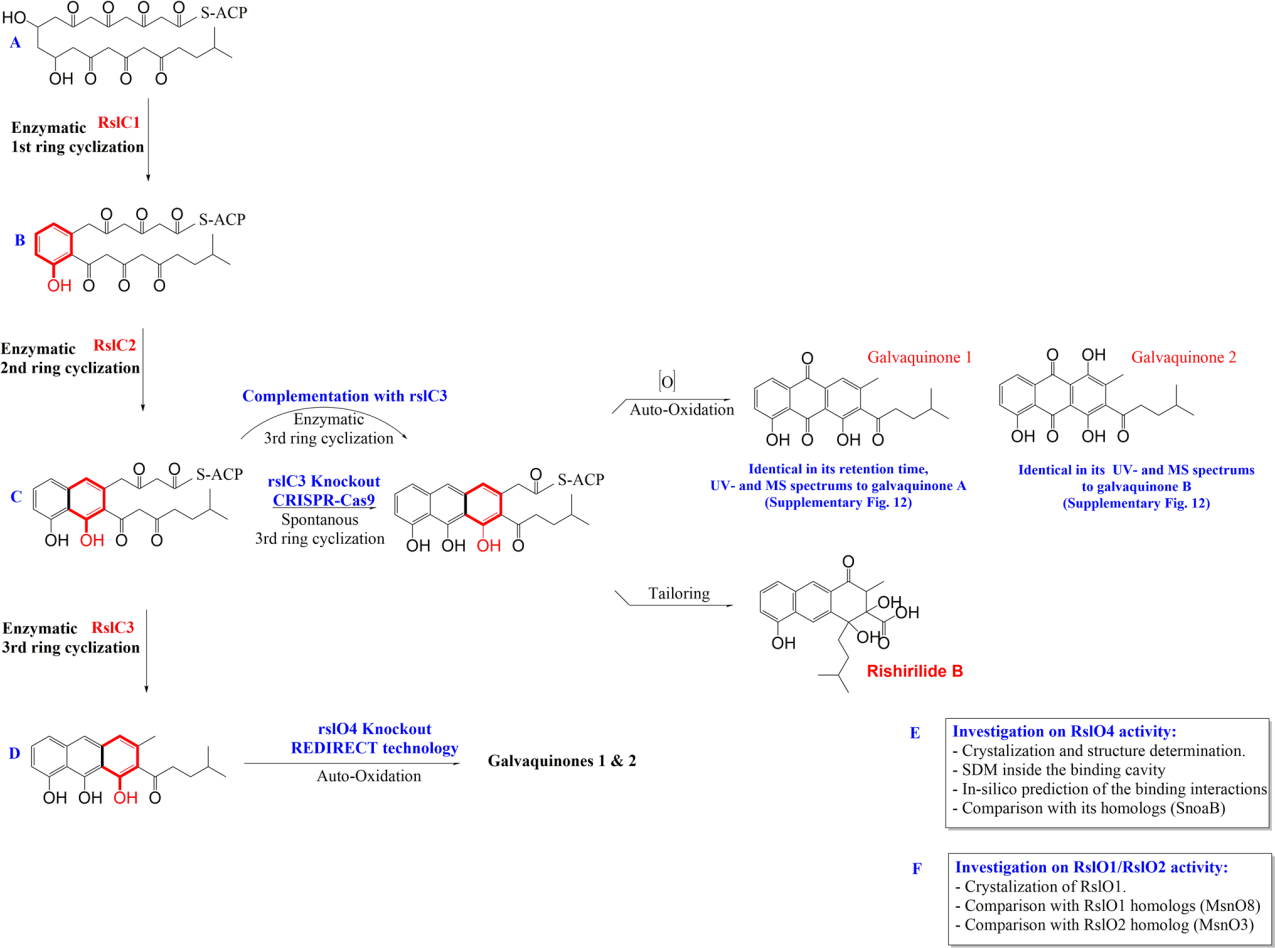
Flow chart of methods and results obtained in this study: **a** Based on sequence comparison RslC1 is responsible for the enzymatic first ring cyclization/aromatization in rishirilide biosynthesis and **b** RslC2 is responsible for the enzymatic second ring cyclization/aromatization in rishirilide biosynthesis. **c** Knockout of 3rd ring cyclase gene “*rslC3*” using CRISPR-Cas9 system resulted in the production of small amounts of galvaquinone 1 and 2. Complementation with *rslC3* gene resulted in the reproduction of rishirilide B. **d** Knockout of the monooxygenase “*rslO4*” using REDIRECT® technology resulted in the production of small amount of galvaquinones 1 and 2. **e** Studies on RslO4. **f** Studies on RslO1/RslO2

Protein sequence alignment of RslO2 with MsnO3 revealed a high identity (47%) using BLASTp. MsnO3 acts as flavin reductase by NADH to provide MsnO8 with the essential reduced FMN for epoxidation and conversion of deoxymensacarcin to mensacarcin [42]. A summary of results obtained in this study is given in Fig. 6.

## Discussion

In our previous studies about rishirilide biosynthesis we could show that RslK4 and RslO3 are involved in the selection of the starter unit. The assembly of rishirilide polyketone from 9 acetate units and one valine-derived isobutyrate is mediated by the minimal PKSs (RslK1, RslK2, RslK3) and RslA [4, 5]. It was proposed that RslO10 acts as C9-ketoreductase and supports the first ring cyclization in rishirilide polyketone [5].

Sequence analysis of *rslC1*, *rslC2* and *rslC3* suggests that they act as cyclases during rishirilide biosynthesis. Homology of RslC1 (SnoaE 53%, RdmK 54% and ChaF 55%) is responsible for 1st ring cyclization in nogalomycin-, rhodomycin- and chartereusin-biosynthesis, respectively [18, 19]. We therefore propose that RslC1 and RslO10 mediate the cyclization/aromatization of the 1st ring in rishirilide-biosynthesis. RslC2 homologs (ActIV 55%, AlnR 54% and Ssfy2 53%) act as 2nd ring cyclases during actinorhodin-, alnumycin- and tetracycline-biosynthesis, respectively [20–22]. Thus RslC2 is considered as 2nd cyclase in rishirilide biosynthesis. And homologs of RslC3 (Cmm 49%, Oxy 44% and SnoO 50%) are involved in ring cyclization of the last ring during the biosynthesis of chromomycin A3, oxytetracycline and nogalomycin, respectively. Thus RslC3 is most likely the 3^rd^ ring cyclase involved in rishirilide biosynthesis [23–25].

A mutant with a deletion in *rslC3* was still producing small amount of rishirilide B indicating that the formation of the third ring can also occur non-enzymatically.

The production of galvaquinone 1 (galvaquinone A) and galvaquinone 2 in our mutants indicate that the cyclization step 3, catalyzed by RslC3, also occurs chemically, although to a much smaller extend. It also indicates that early intermediates of the pathway chemically easily convert to Galvaquinone derivatives. (Supplementary Fig. S1).

The deep-sea-derived *S. olivaceus* SCSIO T05 was described to produce rishirilides and galvaquinone derivatives. The rishirilide cluster (rsd-cluster) in this strain is very similar to the rsl-cluster in *S. bottropensis*. [2]. And recently the rishirilide derivative Lupinacidin A and Galvaquinone B have been isolated from the sea anemone Gyractis sesere [43]. These results indicate that intermediates of biosynthetic pathways to rishirilides can chemically be converted efficiently to galvaquinones.

As we were not able to detect novel intermediates of the pathway in our mutants we decided to focus our research on RslO4 and RslO1, which are both involved in biosynthetic steps which follow the cyclization process.

The high similarity of RslO4 in the sequence and three-dimensional structure with the monooxygenase SnoaB suggests that RslO4 mediates a mono-oxygenation in rishirilide biosynthetic pathway. Interestingly, complementation of *S. albus* × Cos4Δ*rslO4* with *rslO4* restored rishirilide production but complementation with *snoaB* did not indicate different function of both enzymes. Unlike SnoaB which oxygenates the middle non-aromatic ring of 12-deoxynogalonic acid during nogalomycin biosynthesis [27], RslO4 might oxygenate the third lateral aromatic ring in its tricyclic aromatic substrate to form a quinonic product.

**Fig. 7:**
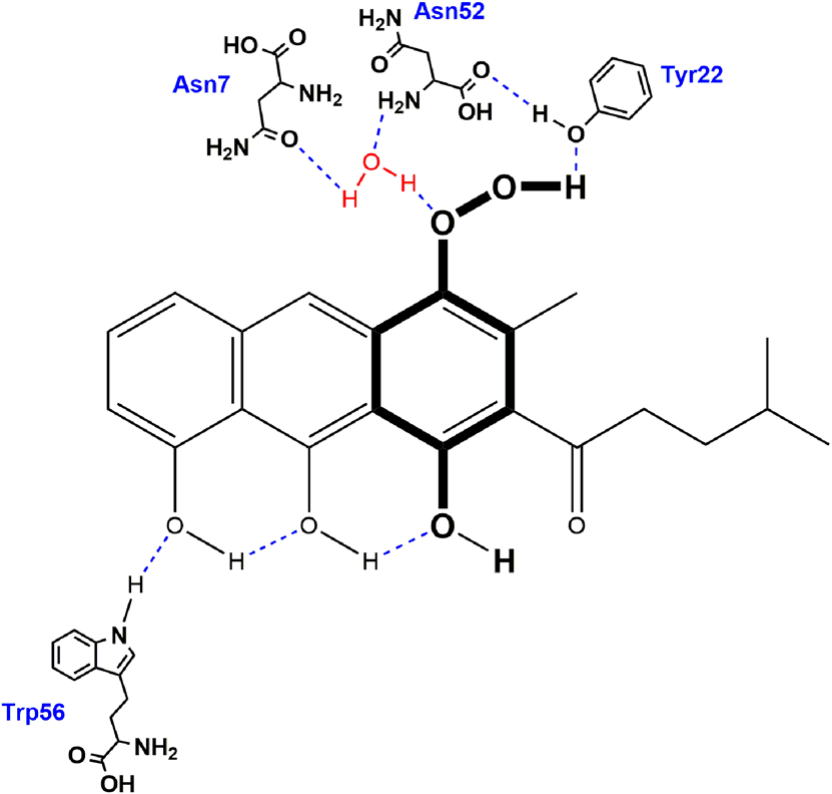
Proposed binding of a putative substrate in the binding pocket of RslO4

According to the observation from molecular dynamics simulations, Trp56 contributes in the orientation of substrate positioning inside RslO4 pocket through an H-Bonding network with the three –OH groups, which in turn leads to an electron delocalization in the third aromatic ring. It is possible that Asn7-Asn52-Wat3 triad catalyzes a proton abstraction from the tricyclic aromatic substrate and reduces the activated peroxide with the same proton to form a peroxy intermediate (Fig. 7).

This intermediate is stabilized inside RslO4 binding center by Asn7-Asn52-Wat3 triad and Trp56, in addition to Tyr22 through hydrogen bindings. The results of dynamic simulations match those of the Site-Directed Mutagenesis which demonstrated the importance of Asn7, Asn52, Trp56, Tyr22, and Met29 in the catalytic mechanism of RslO4. Although the computer simulations did not indicate any cooperation of Met29 in the reaction, it is conceivable that this residue helps in the protein conformational stability.

**Fig. 8:**
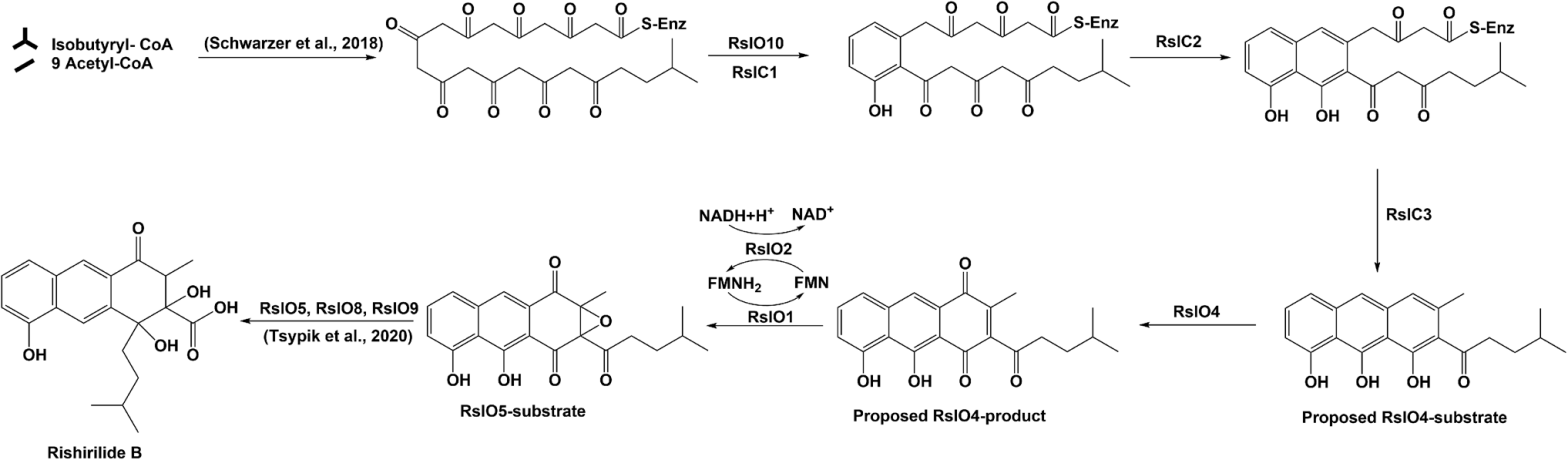
Proposed Biosynthetic pathway of rishirilide B

The present mechanism seems to be consistent with other research which found that Trp67 in SnoaB and Trp66 in ActVA-Orf6 are mainly responsible for the orientation and electron delocalization in their substrates. There are similarities between the roles of Asn7-Asn52-Wat3 triad in RslO4 and the role of Asn18-Asn63-Wat33 in SnoaB. In accordance with the contribution of Tyr51 in stabilizing the peroxy intermediate of ActVA-Orf6, Tyr22 seems to be important for the stabilization of RslO4 intermediate. Following oxygenation, this intermediate would release H_2_O to form RslO4 quinone product [27, 32, 44] (Fig. 8).

RslO1 belongs to the family of FMN-dependent luciferase-like monooxygenases. The high similarity in three-dimensional structure between RslO1 and the epoxy-forming MsnO8 enzyme in mensacarcin biosynthesis in *S. bottropensis* indicates a similar activity of RslO1 enzyme in the epoxide formation to form RslO5-substrate during rishirilide biosynthesis in the same strain. According to the high sequence identity between RslO2 and MsnO3 (47%), it is most likely that RslO2 acts as flavin reductase, by using NADH as a hydrogen source, to provide RslO1 with the reduced FMN in a similar mechanism of MsnO8-MsnO3 two-component epoxide-forming system [42] (Fig. 8).

Following the epoxidation mediated by RslO1-RslO2 system, RslO1 product undergoes an epoxide reduction by RslO5 and then oxidative carbon backbone rearrangement including RslO9-mediated Baeyer–Villiger oxidation and lactone formation. Subsequently, RslO8 catalyzes a keto-reduction to form rishirilide A. Lactone-opening and water elimination in rishirilide A leads to the production of rishirilide B [6] (Fig. 8).

## Conclusion

During rishirilide biosynthesis, RslC3 mediates a cyclization step leading to an intermediate with an unknown structure. The cofactor-independent monooxygenase RslO4 catalyzes the quinone formation of the third ring. The putative flavin reductase RslO2 uses NADH as a source of hydrogen and provides RslO1 with the reduced FMN. In his turn the FMN-dependent luciferase-like monooxygenase RslO1 is responsible for the formation of an epoxide providing a substrate for RslO5.

## Supplementary Information

Below is the link to the electronic supplementary material.

Supplementary file1 (DOCX 2420 KB)

## Abbreviations

Rsl gene cluster: Rishirilide gene cluster
PKS II: Polyketide Synthase type II
CRISPR-Cas9: Clustered Regularly Interspaced Short Palindromic Repeats system
FMN: Flavinmononucleotide
HPLC/ESI–MS: High-Performance Liquid Chromatography/Electrospray Ionization
IMAC Ni–NTA: Immobilized Metal Affinity Chromatography Nickle-Nitrilotriacetic Acid
PDB: Protein Data Bank
AUC: Area Under the Curve
CYC/ARO: Cyclase/Aromatase
SDS-PAGE: Sodium Dodecyl Sulphate–Polyacrylamide Gel Electrophoresis
His-Tag: Histidine tag
NAD: Nicotinamide Adenine Dinucleotide
Apramycin^R^: Apramycin Resistance
Spectinomycin^R^: Spectinomycin Resistance
SDM: Site-Directed Mutagenesis
IPTG: Isopropyl β-D-1-thiogalactopyranoside

## References

[1] Iwaki H, Nakayama Y, Takahashi M, Uetsuki S, Kido M, Fukuyama Y (1984). Structures of rishirilides A and B, α2-macroglobulin inhibitors produced by *Streptomyces rishiriensis* OFR-1056. Journal of Antibiotics.

[2] Zhang C, Sun C, Huang H, Gui C, Wang L, Li Q, Ju J (2018). Biosynthetic baeyer−villiger chemistry enables access to two anthracene scaffolds from a single gene cluster in deep-sea-derived *Streptomyces olivaceus* SCSIO T05. Journal of Natural Products.

[3] Odagi M, Furukari K, Takayama K, Noguchi K, Nagasawa K (2017). Total synthesis of rishirilide B by organocatalytic oxidative kinetic resolution: Revision of absolute configuration of (+)-Rishirilide B. Angewandte Chemie International Edition.

[4] Schwarzer P, Wunsch-Palasis J, Bechthold A, Paululat T (2018). Biosynthesis of rishirilide B. Antibiotics.

[5] Schwarzer P, Tsypik O, Zuo C, Alali A, Wunsch-Palasis J, Heitzler T, Derochefort J, Bernhardt M, Yan X, Paululat T, Bechthold A (2020). Early steps in the biosynthetic pathway of rishirilide B. Molecules.

[6] Tsypik O, Makitrynskyy R, Frensch B, Zechel DL, Paululat T, Teufel R, Bechthold A (2020). Oxidative carbon backbone rearrangement in rishirilide biosynthesis. Journal of the American Chemical Society.

[7] Tong Y, Charusanti P, Zhang L, Weber T, Lee SY (2015). CRISPR-Cas9 based engineering of actinomycetal genomes. ACS Synthetic Biology.

[8] Gust B, Kieser T, Chater KF (2002). REDIRECT© technology: PCR-targeting system in *Streptomyces coelicolor*.

[9] Battye TGG, Kontogiannis L, Johnson O, Powell HR, Leslie AGW (2010). iMOSFLM: A new graphical interface for diffraction image processing with MOSFLM. Acta Cryst..

[10] Kabsch W (2009). XDS. Acta Crystallographica.

[11] Keegan RM, Winn MD (2007). MrBUMP: an automated pipeline for molecular replacement. Acta Crystallographica.

[12] Cowtan K (2006). The *Buccaneer* software for automated model building. 1. Tracing protein chains. Acta Crystallographica.

[13] Emsley P, Cowtan K (2004). Coot: Model-building tools for molecular graphics. Acta Crystallographica.

[14] Afonine PV, Grosse-Kunstleve RW, Echols N, Headd JJ, Moriarty NW, Mustyakimov M, Terwilliger TC, Urzhumtsev A, Zwart PH, Adams PD (2011). Towards automated crystallographic structure refinement with phenix.refine. Acta Crystallographica.

[15] Chen VB, Arendall WB, Headd JJ, Keedy DA, Immormino RM, Kapral GJ, Murray LW, Richardson JS, Richardson DC (2009). MolProbity: All-atom structure validation for macromolecular crystallography. Acta Crystallographica.

[16] Vonrhein C, Flensburg C, Keller P, Sharff A, Smart O, Paciorek W, Womack T, Bricogne G (2010). Data processing and analysis with the autoPROC toolbox. Acta Crystallographica.

[17] Vagin A, Teplyakov A (2009). Molecular replacement with MOLREP. Acta Crystallographica.

[18] Xu Z, Jakobi K, Welzel K, Hertweck C (2005). Biosynthesis of the antitumor agent chartreusin involves the oxidative rearrangement of an anthracyclic polyketide. Chemistry & Biology.

[19] Klymyshin DA, Stefanyshyn ON, Fedorenko VA (2015). Role of genes *snoaM*, *snoaL*, and *snoaE* in the biosynthesis of nogalamycin in *Streptomyces nogalater* Lv65. Cytology and Genetics.

[20] Taguchi T, Awakawa T, Nishihara Y, Kawamura M, Ohnishi Y, Ichinose K (2017). Bifunctionality of ActIV as a cyclase-thioesterase revealed by in vitro reconstitution of actinorhodin biosynthesis in *Streptomyces coelicolor* A3(2). ChemBioChem.

[21] Oja T, Palmu K, Lehmussola H, Leppäranta O, Hännikäinen K, Niemi J, Mäntsälä P, Metsä-Ketelä M (2008). Characterization of the alnumycin gene cluster reveals unusual gene products for pyran ring formation and dioxan biosynthesis. Chemistry & Biology.

[22] Pickens LB, Kim W, Wang P, Zhou H, Watanabe K, Gomi S, Tang Y (2009). Biochemical analysis of the biosynthetic pathway of an anticancer tetracycline SF2575. Journal of the American Chemical Society.

[23] Menendez N, Nur-e-Alam M, Brana AF, Rohr J, Salas JA, Mendez C (2004). Biosynthesis of the antitumor chromomycin A3 in *Streptomyces griseus*: Analysis of the gene cluster and rational design of novel chromomycin analogs. Chemistry & Biology.

[24] Petković H, Lukežič T, Šušković J (2017). Biosynthesis of oxytetracycline by *Streptomyces rimosus*: Past, present and future directions in the development of tetracycline antibiotics. Food Technology and Biotechnology.

[25] Gullon S, Olano C, Abdelfattah MS, Brana AF, Rohr J, Mendez C, Salas JA (2006). Isolation, characterization, and heterologous expression of the biosynthesis gene cluster for the antitumor anthracycline steffimycin. Applied and Environment Microbiology.

[26] Waterhouse A, Bertoni M, Bienert S, Studer G, Tauriello G, Gumienny R, Heer FT, de Beer TAP, Rempfer C, Bordoli L, Lepore R, Schwede T (2018). SWISS-MODEL: Homology modelling of protein structures and complexes. Nucleic Acids Research.

[27] Grocholski T, Koskiniemi H, Lindqvist Y, Mäntsälä P, Niemi J, Schneider G (2010). Crystal structure of the cofactor-independent monooxygenase SnoaB from *Streptomyces nogalater*: Implications for the reaction mechanism. Biochemistry.

[28] Rezacova P, Borek D, Moy SE, Joachimiak A, Otwinowski Z (2008). Crystal structure and putative function of small Toprim domain-containing protein from *Bacillus stearothermophilus*. Proteins.

[29] Sanders DAR, Walker JR, Skarina T, Savchenko A (2005). The X-ray crystal structure of PA3566 from *Pseudomonas aureginosa* at 1.8 Å resolution. Proteins.

[30] Wada T, Shirouzu M, Terada T, Kamewari Y, Park SY, Tame JRH, Kuramitsu S, Yokoyama S (2004). Crystal structure of the conserved hypothetical protein TT1380 from *Thermus thermophilus* HB8. Proteins.

[31] Chao A, Goulding CW (2019). A single mutation in the *Mycobacterium tuberculosis* heme-degrading protein, MhuD, results in different products. Biochemistry.

[32] Sciara G, Kendrew SG, Miele AE, Marsh NG, Federici L, Malatesta F, Schimperna G, Savino C, Vallone B (2003). The structure of ActVA-Orf6, a novel type of monooxygenase involved in actinorhodin biosynthesis. The EMBO Journal.

[33] Larkin MA, Blackshields G, Brown NP, Chenna R, McGettigan PA, McWilliam H, Valentin F, Wallace IM, Wilm A, Lopez R, Thompson JD, Gibson TJ, Higgins DG (2007). Clustal W and Clustal X version 2.0. Bioinformatics.

[34] Robert X, Gouet P (2014). Deciphering key features in protein structures with the new ENDscript server. Nucleic Acids Research.

[35] Li L, Liu X, Yang W, Xu F, Wang W, Feng L, Bartlam M, Wang L, Rao Z (2007). Crystal structure of long-chain alkane monooxygenase (LadA) in complex with coenzyme FMN: Unveiling the long-chain alkane hydroxylase. Journal of Molecular Biology.

[36] Shima S, Warkentin E, Grabarse W, Sordel M, Wicke M, Thauer RK, Ermler U (2000). Structure of coenzyme F420 dependent methylenetetrahydromethanopterin reductase from two methanogenic archaea. Journal of Molecular Biology.

[37] Oyugi MA, Bashiri G, Baker EN, Johnson-Winters K (2016). Investigating the reaction mechanism of F420-dependent glucose-6-phosphate dehydrogenase from *Mycobacterium tuberculosis*: Kinetic analysis of the wild-type and mutant enzymes. Biochemistry.

[38] Bashiri G, Squire CJ, Moreland NJ, Baker EN (2008). Crystal structures of F420-dependent glucose-6-phosphate dehydrogenase FGD1 involved in the activation of the anti-tuberculosis drug candidate PA-824 reveal the basis of coenzyme and substrate binding. Journal of Biological Chemistry.

[39] Greening C, Ahmed FH, Mohamed AE, Lee BM, Pandey G, Warden AC, Scott C, Oakeshott JG, Taylor MC, Jackson CJ (2016). Physiology, biochemistry, and applications of F420- and Fo-dependent redox reactions. Microbiology and Molecular Biology Reviews.

[40] Eichhorn E, Davey CA, Sargent DF, Leisinger T, Richmond TJ (2002). Crystal structure of *Escherichia coli* alkanesulfonate monooxygenase SsuD. Journal of Molecular Biology.

[41] Campbell ZT, Weichsel A, Montfort WR, Baldwin TO (2009). Crystal structure of the bacterial luciferase/flavin complex provides insight into the function of the *β*Subunit. Biochemistry.

[42] Maier S, Pflüger T, Loesgen S, Asmus K, Brötz E, Paululat T, Zeeck A, Andrade S, Bechthold A (2014). Insights into the bioactivity of mensacarcin and epoxide formation by MsnO8. ChemBioChem.

[43] Sottorff I, Künzel S, Wiese J, Lipfert M, Preußke N, Sönnichsen FD, Imhoff JF (2019). Antitumor anthraquinones from an Easter Island Sea Anemone: animal or bacterial origin?. Marine Drugs.

[44] Okamoto S, Taguchi T, Ochi K, Ichinose K (2009). Biosynthesis of actinorhodin and related antibiotics: Discovery of alternative routes for quinone formation encoded in the act gene cluster. Chemistry & Biology.

