# Supplementary material for: Biosynthesis of the Tricyclic Aromatic Type II Polyketide Rishirilide: New Potential Third Ring Oxygenation after Three Cyclization Steps
Source: Mol Biotechnol. 2021 Mar 24;63(6):502–14. doi: 10.1007/s12033-021-00314-x (PMC8093152; doi:10.1007/s12033-021-00314-x)
Supplement: Supplementary file 1 — Supplementary file1 (DOCX 2420 KB) [file 12033_2021_314_MOESM1_ESM.docx]

**Molecular Biotechnology**

**Biosynthesis of the Tricyclic Aromatic type II Polyketide Rishirilide:**

**New Potential Third Ring Oxygenation after Three Cyclization Steps.**

**^1^Ahmad Alali, ^2^Lin Zhang, ^3^Jianyu Li, ^1^Chijian Zuo, ^1^Dimah Wassouf, ^1^Xiaohui Yan, ^1^Philipp Schwarzer, ^3^Stefan Günther, ^2^Oliver Einsle and ^1^Andreas Bechthold.**

**Corresponding Author:**

Prof. Dr. Andreas Bechthold

Institute of Pharmaceutical Biology and Biotechnology, Freiburg, Germany.
Email: [andreas.bechthold@pharmazie.uni-freiburg.de](mailto:andreas.bechthold@pharmazie.uni-freiburg.de)

^1^ Institute of Pharmaceutical Biology and Biotechnology, Albert-Ludwigs-Universität, Stefan-Meier-Straße 19, 79104 Freiburg, Germany

^2^ Institute of Biochemistry, Albert-Ludwigs-Universität, Albertstr 21, 79104 Freiburg, Germany

^3^ Institute of Pharmaceutical Bioinformatics, Albert-Ludwigs-Universität, Hermann-Herder-Str 9, 79104 Freiburg, Germany


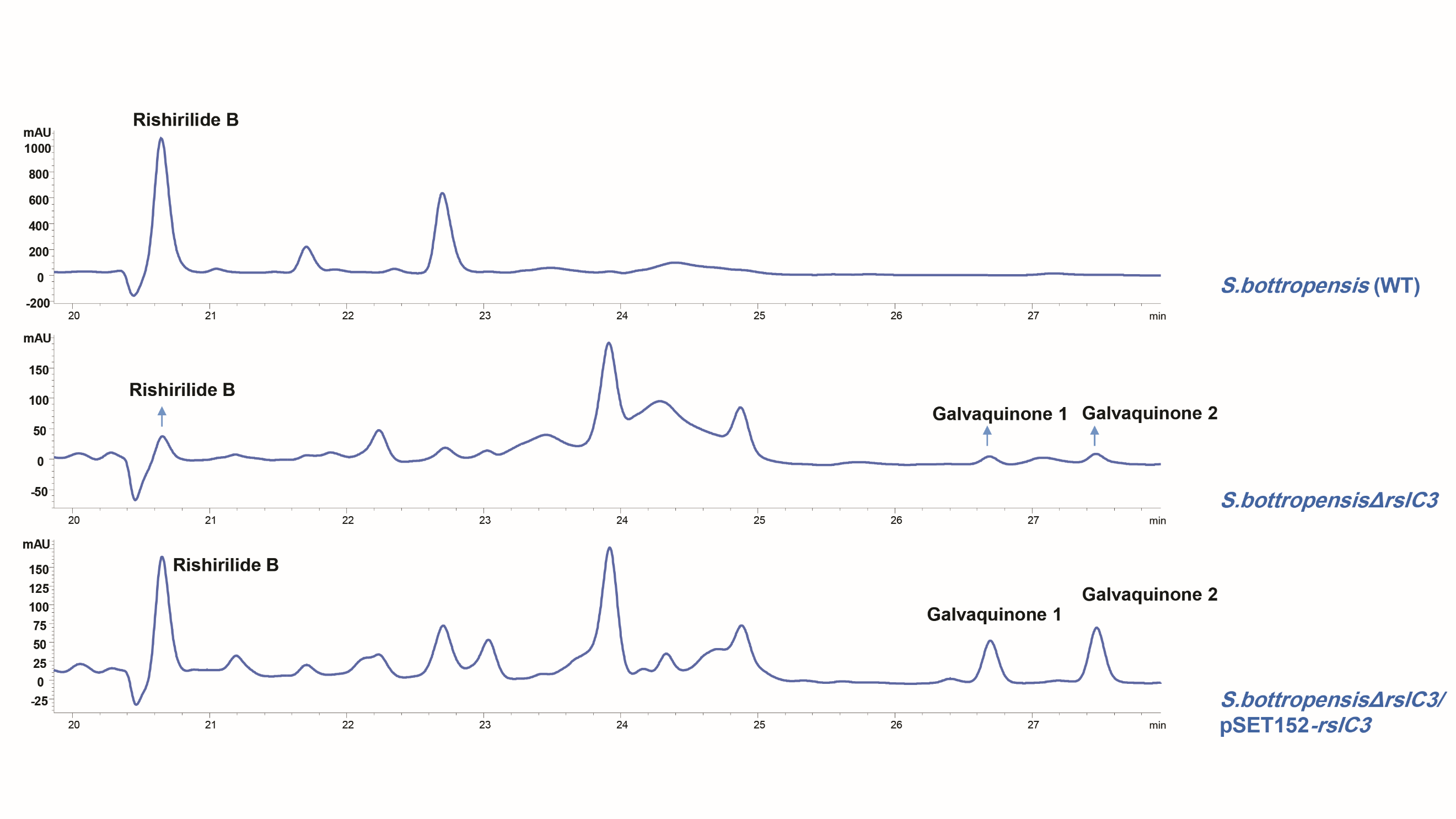


**Fig.S1.** **HPLC-Chromatograms (λ=254nm) obtained from, HPLC/ESI-MS analysis of S.bottropensis (WT), S.bottropensisΔrslC3, and S.bottropensisΔrslC3/pSET152-rslC3 crude extracts:** In the wide type strain, rishirilide B is produced in significant amounts. Rishirilide B is produced in trace amount after gene inactivation of rslC3 and galvaquinones (1, 2) can be detected in small amounts. Complementation with rslC3 resulted in reproducing rishirilide B and increasing the production of galvaquinones (1, 2).


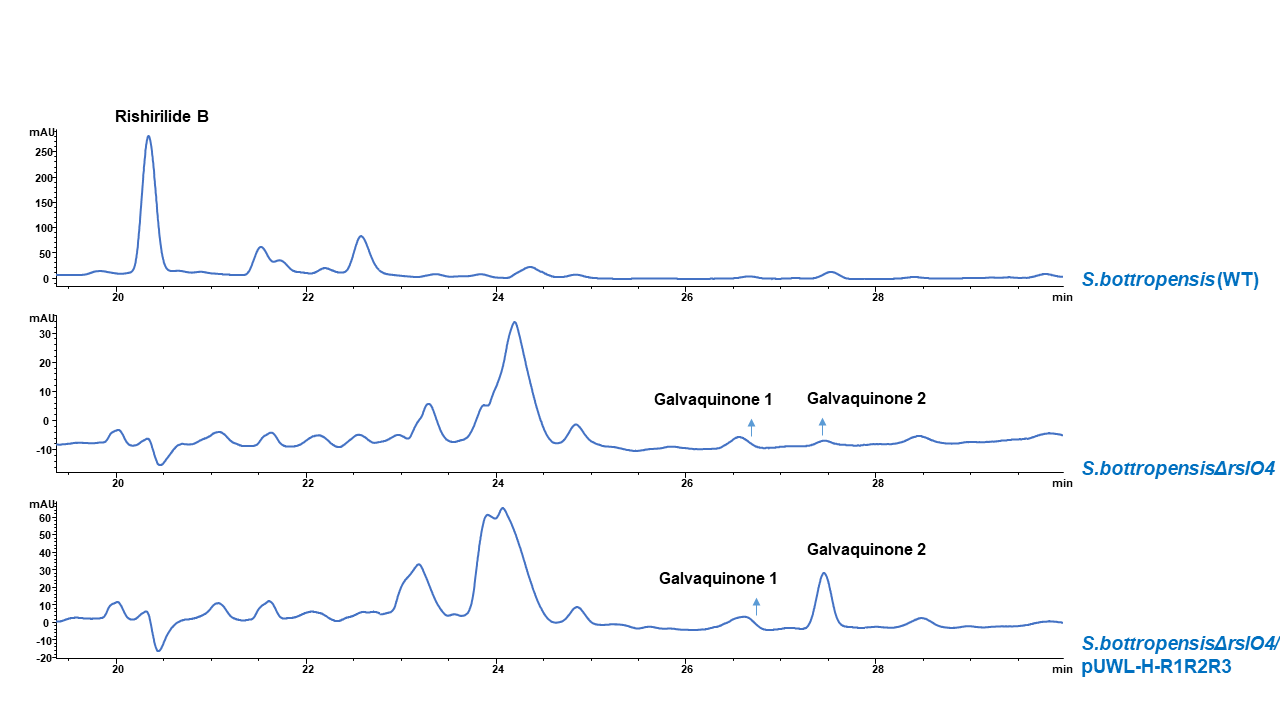


**Fig.S2.** **HPLC-Chromatograms (λ=254nm) obtained from, HPLC/ESI-MS analysis of S.bottropensis (WT), S.bottropensisΔrslO4, and S.bottropensisΔrslO4/pUWL-H-rslR1R2R3 crude extracts:** In the wide type strain, rishirilide B is produced in significant amounts. Biosynthesis of rishirilide B is totally interrupted by rslO4 inactivation. Production of galvaquinone 2 can increase significantly by the induction with positive regulators rslR1, rslR2 and rslR3. Using MS-Spectrum, induction of galvaquinone 1 prodcution was detected in trace amount by the positive regulators. The natural substrate of RslO4 can not be found.


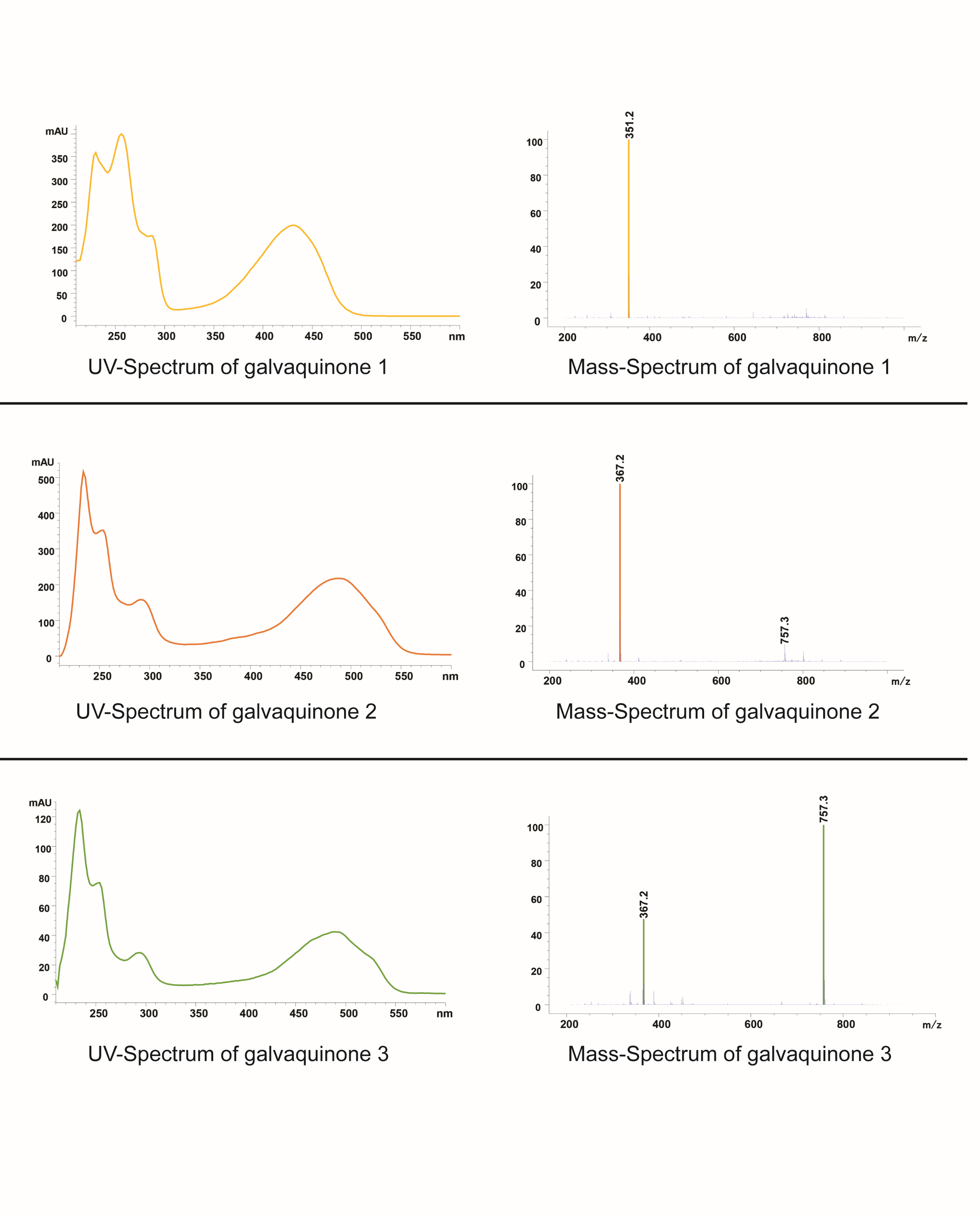


**Fig.S3.** **UV- and Mass-spectrums of galvaquinones obtained from HPLC/ESI-MS S.bottropensisΔrslC3 (galvaquinone 1 and 2, Fig. S1):** galvaquinone 1 has identical UV- and Mass spectrums of galvaquinone A, galvaquinone 2 have identical UV- and Mass spectrums of galvaquinone B.


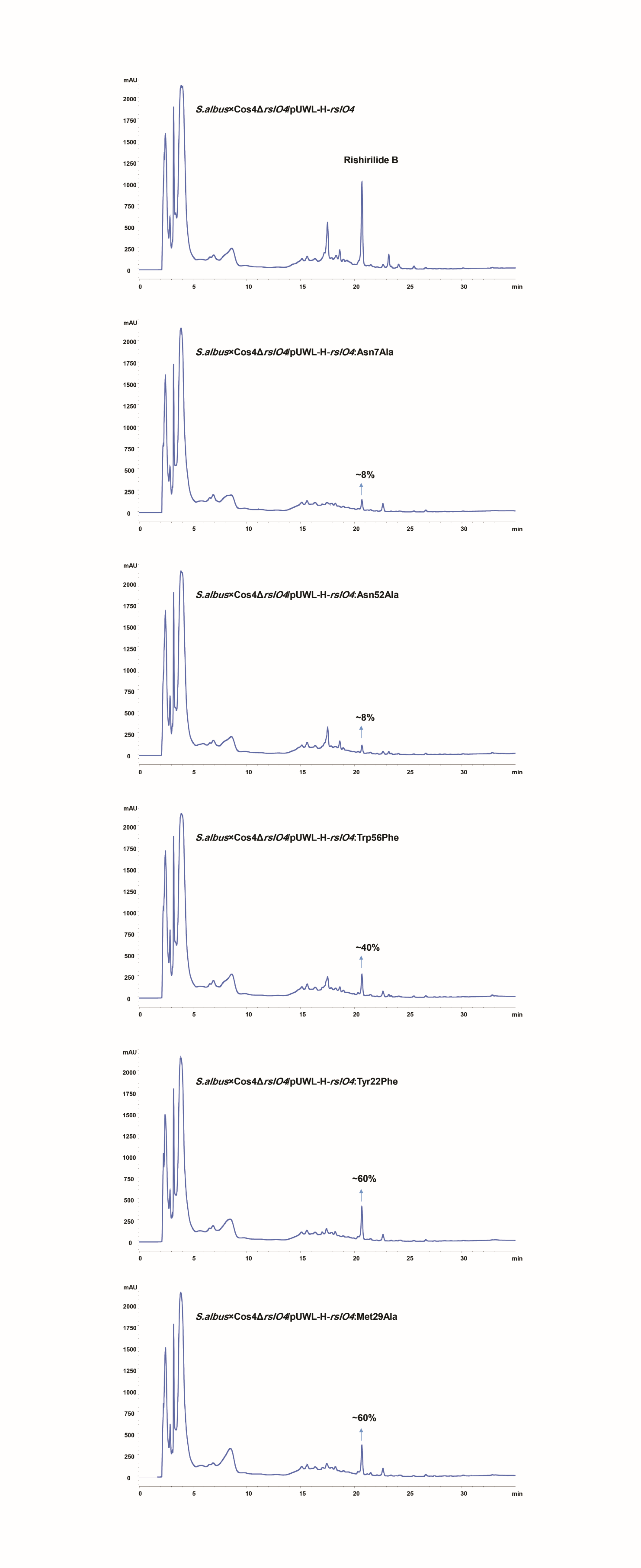


**Fig.S4. Site-directed mutagenesis (SDM) of** **RslO4 active site residues:** complementation experiments in *S.albus*×Cos4Δ*rslO4* with mutated *rslO4* gene cloned in pUWL-H plasmid showed approximately 8% rishirilide production after the replacement of Asn7 and Asn52 with Ala, 40% after the replacement of Trp56 with Phe, 60% after the replacement of Tyr22 and Met29 with Phe and Ala, respectively.

**Supplementary Table. 1: Primers used for this work**

| **Primers used for CRISPR-Cas9 system:** | | |
| --- | --- | --- |
| **Primer** | **Sequence** | **Notes** |
|  |  | For amplifying sgRNA targeting *rslC3*.  For PCR verification of sgRNA cloning with CRISPR-Cas9 vector.  C3-sgRNA-R was used for sequencing verification of the successful cloning. |
|  |  |  |
| C3-sgRNA-F | CATGCCATGGTCATGCCGAACCAGT  GGCGGGTTTTAGAGCTAGAAATAGC |  |
| C3-sgRNA-R | ACGCCTACGTAAAAAAAGCACCGAC  TCGGTGCC |  |
| rslC3-mut-detect-F | TTGAGGTGTTCGCCGGGCTC | For PCR verification of *rslC3* knockout by annealing 200bps up- and downstream *rslC3* gene. |
| rslC3-mut-detect-R | GCCGACTACCTTGGTGATCTCG |  |
|  |  |  |
| C3-Tem5-1 | CGAAGCACAGGAGGAAGACCGCAT  GAGCGGCCTGTTCGGC | To amplify downstream template of *rslC3*.  C3-Tem5-2 was used for overlapping PCR. |
| C3-Tem5-2 | GAGGGCCGGATCAVTGCCCGCGAC  GAAGTG |  |
| C3-Tem3-1 | GCCGAACAGGCCGCTCATGCGGTCT  TCCTCCTGTGCTTCG | To amplify upstream template of *rslC3*.  C3-Tem3-2 was used for overlapping PCR. |
| C3 -Tem3-2 | GACGAACTGTGGGCGGACTCGCCCC  CGCACAG |  |
| C3-Comp-F | GGAGTCTCGCCGTGCAGGTC | To amplify *rslC3* gene for the complementation experiment in *S.bottropensis*Δ*rslC3* using pSET152 vector. |
| C3-Comp-R | CGACTCTAGACGCGAGTCGGAGCTC  CTG |  |
| **Primers used in Redirect® technology for the construction of non-integrative *rslO4*-mutant Cos4×spec:** | | |
| **Primer** | **Sequence** | **Notes** |
| Cos4(Apra-to-Spec)-F | CTATTTGCAACAGTGCCGTTG  ATCGTGCTATGATCGACTGAT  GAGGGAAGCGGTGATCGCC | To amplify spectinomycin^R^ cassette for replacement of apramycin^R^ marker in non-integrative Cos4 by homologous recombination. |
| Cos4(Apra-to-Spec)-R | ACCTTGCCCCTCCAACGTCAT  CTCGTTCTCCGCTCATGAGCT  TATTTGCCGACTACCTTGGT |  |
| Spec-detect-F | ATGAGGGAAGCGGTGATCGC  CGAA | For PCR verification of the replacement of apramycin^R^ marker with spectinomycin^R^ cassette in non-integrative Cos4. |
| Spec-detect-R | ATTTGCCGACTACCTTGGTG |  |
| Spec-Cos-Seq | GGTTCATGTGCAGCTCCATC | For sequencing verification of the replacement of apramycin^R^ marker with spectinomycin^R^ cassette. |
| O4-apra-F | ACGACCACCCGCGTACGAACA  TCCACCCGAGGAGACCACCAT  GAACAAAAGCTGGAGCTC | To amplify apramycin^R^ cassette for replacement of *rslO4* gene in non-integrative Cos4×Spec by homologous recombination. |
| O4-apra-R | CGGCGCGTGGTGTACGTGCCG  GCCCGCGGTCCGCTCCGGCTC  AGATATCTCTAGATACCG |  |
| rslO4(mut-det)F | CGGGGTGACCGTGAACTGCG | For PCR verification of the replacement of *rslO4* gene with apramycin^R^ cassette in *S.bottropensis*. |
| rslO4(mut-det)R | TCGTCAACCTCGGCACCGGA |  |
| **Primers used for C-terminal His-tag recombinant RslO1 and RslO4 protein production:** | | |
| **Primer** | **Sequence** | **Notes** |
| RslO1(21a)-F | GCGCCATATGAAGTTCGGCATCAACCT  CTTCCC | To amplify *rslO1* gene for cloning with the expression vector pET21a(+) between NdeI and XhoI restriction sites. |
| RslO1(21a)-R | CCGCTCGAGGTCGTTCGCTGCGTAGTG |  |
| RslO4(21a)-F | GCGCCATATGGCCGTCGTGTTCGTCAA | To amplify *rslO4* gene for cloning with the expression vector pET21a(+)between NdeI and XhoI restriction sites. |
| RslO4(21a)-R | TATACTCGAGGACCTGGGCGGCCCGG |  |
| **Primers used for in vivo comparison of RslO4 and SnoaB activities:** | | |
| **Primer** | **Sequence** | **Notes** |
| rslO4-f(ClaI) | CCATCGATAAGTCCGCCCCACGTACCCGC | For amplification of *rslO4.* |
| rslO4-r(SpeI) | GGACTAGTGCGGTCCGCTCCGGCTCAGA |  |
| snoaB-f (ClaI) | AAAATCGATCGTACGGACCCACACCACA | For amplification of *snoaB.* |
| snoaB-r(SpeI) | AAAACTAGTCCTCAGCGATGTCCGGAG |  |
| **Primers used for Side-Directed Mutagenesis (SDM) inside the binding cavity of RslO4 protein:** | | |
| **Primer** | **Sequence** | **Notes** |
| Asn7-F | (Pho)AGCGACGAACACGACGGCCATGG  TGGTCTC | For amplification of pUWL-H-*rslO4* with substitution mutation in *rslO4* sequence (asparagine 7 to alanine). |
| Asn7-R | (Pho)AAGCTCACCCTGATCGGCGACGCC  GAGGAGTTC |  |
| Tyr22-F | (Pho)GAAGCGGCTCTCGAACTCCTCGGCGTC | For amplification of pUWL-H-*rslO4* with substitution mutation in *rslO4* sequence (tyrosine 22 to phenylalanine). |
| Tyr22-R | (Pho)GAGGCCGTCGGAGCCTTCATGGAG |  |
| Met29-F | (Pho)AGCGAAGGCTCCGACGGCCTCGTA | For amplification of pUWL-H-*rslO4* with substitution mutation in *rslO4* sequence (methionine 29 to alanine). |
| Met29-R | (Pho)GAGACCCAGCCGGGCCTCGTCC |  |
| Asn52-F | (Pho)AGCGAAGTACACGGAGTCGTCCTTGG | For amplification of pUWL-H-*rslO4* with substitution mutation in *rslO4* sequence (asparagine 52 to alanine). |
| Asn52-R | (Pho)ATCGCCGAGTGGGATGACGAGGACAC  CTTC |  |
| Trp56-F | (Pho)GAACTCGGCGATGTTGAAGTACACG | For amplification of pUWL-H-*rslO4* with substitution mutation in *rslO4* sequence (tryptophan 56 to phenylalanine). |
| Trp56-R | (Pho)GATGACGAGGACACCTTCCGCAAGG |  |

**Supplementary Table. 2: Parameters and methods used for the analytical HPLC ESI/MS**

| **Analytic HPLC ESI/MS parameter:** | | |
| --- | --- | --- |
| **Parameter** | **Settings** | |
| Solvent A | Acetonitrile + 0.5% acetic acid *(V/V)* | |
| Solvent B | H_2_O + 0.5% acetic acid *(V/V)* | |
| Flow rate | 0.5 mL/min | |
| Column Temperature | 30ºC | |
| Detection | 254nm (Ref. 400), 230nm (Ref. 400), 330nm (Ref. 500), 400nm (Ref. 600) | |
| MSD Scan | 100 – 500 Da (Neg) | |
| **Analytical HPLC ESI/MS phases according to percentages of solvent A and B for analysis of rishirilide** | | |
| **Time (min)** | **Solvent A (%)** | **Solvent B (%)** |
| 0 | 20 | 80 |
| 6 | 20 | 80 |
| 7 | 30 | 70 |
| 25 | 95 | 5 |
| 28 | 95 | 5 |
| 30 | 20 | 80 |
| 35 | 20 | 80 |
| 0 | 20 | 80 |
| **Parameters of ESI source** | | |
| **Parameter** | **Settings** | |
| Dry gas flow | 12 L/min | |
| Dry gas temperature | 350ºC | |
| Nebulizer pressure | 50 psi | |
| Spray capillary voltage (Vcap pos) | 3000 V | |
| Spray capillary voltage (Vcap neg) | 3000 | |

**Supplementary Table 3: X-ray data collection and refinement statistics**

| **Data sets** | | **RslO1 (PDB ID: 7BIP)** | **RslO4 (PDB ID: 7BIO)** |
| --- | --- | --- | --- |
| Space group | | P 2_1_ 2_1_ 2_1_ | P 3_2_ 2 1 |
| Cell constants | a, b, c [[Å](https://en.wikipedia.org/wiki/%C3%85)]  α, β, γ [°] | 50.91, 81.96, 160.62  90, 90, 90 | 83.7, 83.7, 82.3  90, 90, 120 |
| Wavelength [[Å](https://en.wikipedia.org/wiki/%C3%85)] | | 1 | 1 |
| Resolution limits [[Å](https://en.wikipedia.org/wiki/%C3%85)] | | 48.53 - 1.60 (1.63 - 1.60) | 72.470 – 1.795 (1.912 – 1.795) |
| Completeness (%) | | 100 (99.4) | 94.4 (56.4) |
| Unique reflections | | 89593 (4319) | 25998 (1300) |
| Multiplicity (%) | | 25.8 (23.2) | 19.7 (22.5) |
| *R*_merge_^a^ | | 0.024 (2.446) | 0.056 (2.189) |
| *R*_p.i.m._ | | 0.041 (0.506) | 0.013 (0.471) |
| Mean I/σ (I) | | 13.3 (1.6) | 25.7 (1.4) |
| CC_1/2_ | | 0.999 (0.542) | 1.000 (0.638) |
| **Refinement statistics** | |  |  |
| *R*_work_^b^ / *R*_free_ | | 0.164 / 0.194 | 0.182 / 0.204 |
| No. atoms | | 5783 | 1935 |
| Protein | | 5152 | 1758 |
| Ligand/ion | | 122 | 29 |
| Water | | 509 | 148 |
| B-factor [[Å](https://en.wikipedia.org/wiki/%C3%85)^2^] | | 22.48 | 49.91 |
| Protein | | 21.04 | 49.55 |
| Ligand/ion | | 42.49 | 57.04 |
| Water | | 32.23 | 52.74 |
| R.m.s. deviations | |  |  |
| bond lengths [[Å](https://en.wikipedia.org/wiki/%C3%85)] | | 0.006 | 0.007 |
| bond angels [°] | | 0.81 | 1.21 |
| Ramachandran plot | |  |  |
| Favored (%) | | 96.62 | 99.06 |
| Allowed (%) | | 3.23 | 0.94 |
| Outliers (%) | | 0.15 | 0 |
